# Analytical Model of Eccentric Induction Machines Using the Conformal Winding Tensor Approach

**DOI:** 10.3390/s22093150

**Published:** 2022-04-20

**Authors:** Carla Terron-Santiago, Javier Martinez-Roman, Ruben Puche-Panadero, Angel Sapena-Bano, Jordi Burriel-Valencia, Manuel Pineda-Sanchez

**Affiliations:** Institute for Energy Engineering, Universitat Politècnica de València, Camino de Vera s/n, 46022 Valencia, Spain; cartersa@etsii.upv.es (C.T.-S.); jmroman@die.upv.es (J.M.-R.); rupucpa@die.upv.es (R.P.-P.); asapena@die.upv.es (A.S.-B.); jorburva@die.upv.es (J.B.-V.)

**Keywords:** winding tensor, induction machines, fault diagnosis, mixed eccentricity

## Abstract

Induction machines (IMs) are a critical component of many industrial processes, and their failure can cause large economic losses. Condition-based maintenance systems (CBMs) that are capable of detecting their failures at an incipient stage can reduce these risks by continuously monitoring the IMs’ condition. The development and reliable operations of CBMs systems require rapid modeling of the faulty IM. Due to the fault-induced IM asymmetries, these models are much more complex than those used for a healthy IM. In particular, a mixed eccentricity fault (static and dynamic), which can degenerate into rubbing and destruction of the rotor, produces a non-uniform IM air gap that is different for each rotor position, which makes its very difficult to calculate the IM’s inductance matrix. In this work, a new analytical model of an eccentric IM is presented. It is based on the winding tensor approach, which allows a clear separation between the air gap and winding-related faults. Contrary to previous approaches, where complex expressions have been developed for obtaining mutual inductances between conductors and windings of an eccentric IM, a conformal transformation is proposed in this work, which allows using the simple inductance expressions of a healthy IM. This novel conformal winding tensor approach (CWFA) is theoretically explained and validated with the diagnosis of two commercial IMs with a mixed eccentricity fault.

## 1. Introduction

IMs maintenance is necessary for most industrial process to run smoothly, avoiding unexpected breakdowns of production lines [1,2]. Corrective maintenance can be very costly for companies and is increasingly being replaced by condition-based maintenance systems [3,4,5], which can detect IM faults at an early stage, thus limiting equipment downtime and costs caused by production interruption. Among the different diagnostic techniques proposed in the technical literature to assess the IM condition, motor current signature analysis (MCSA) [6,7,8] has gained an increasing interest, because it is non-invasive and can be implemented with low cost hardware sensors, such as a current clamp, and fast software signal processing tools, such as the fast Fourier transform (FFT).

One of the most common IM failures is rotor eccentricity [9,10]. In this case, the center of rotation of the rotor may not coincide with the axis of symmetry of the stator (static eccentricity), with the axis of symmetry of the rotor (dynamic eccentricity), or with neither of them (mixed eccentricity) [11]. This failure can be produced by the manufacturing process (every IM has an inherent degree of eccentricity) or by working conditions, such as driving an unbalanced load. This produces an unbalanced magnetic pull [12] that can damage bearings and generate abnormal vibrations [13,14] or even cause rotor rubbing, with a total destruction of the machine [15,16]. Therefore, it is very important to detect this fault at an incipient stage so that proper maintenance actions can be scheduled. However, the low amplitude of the fault harmonics generated by an eccentricity fault represents a challenging task for MCSA and requires advanced and fast models of the eccentric IM [17] to develop new signal processing tools or to train artificial intelligent (AI) [18] systems to recognize this type of fault. In this regard, different models of the eccentric IM have been proposed in the technical literature [17,19]:

- Numerical models, mostly based on the finite elements method (FEM). They can accurately reproduce the behaviour of the eccentric IM [20], but they require detailed information about construction aspects of the IM and are computationally intensive. This problem can be alleviated using order-reduction models [21], solving the machine at some positions and performing a field reconstruction based on them or with hybrid FEM-analytical models [22,23,24].
- Analytical models, based on a network of magnetically coupled circuits [25]. Their accuracy may not be as high as FEM models, but they are much faster to build and solve, need only the most basic motor parameters [26], and can correctly reproduce the position and amplitude of the fault-related harmonic components [27].

The main difficulty for developing analytical models of the eccentric IM is the need of an accurate inductance matrix that takes into account the non-uniform air gap length, as a function of the rotor position. This matrix can be built by direct measurement, as in [28,29], or calculated analytically. The winding function approach (WFA) calculates the mutual inductances between different types of phase coils [9,30,31,32] and has been used for IM models in [33,34,35]. However, it requires a numerical solution of definite integrals, which is a product of turn-modified winding and inverse air gap functions, for each rotor position. This is a cumbersome procedure [10], and a simplified model has been proposed in [10], replacing the actual bars of the cage rotor by an equivalent three-phase winding. A different proposal is the winding tensor approach (WTA), which replaces the coil by the conductor as the most basic unit and reduces the calculation process to routine tensor operations [36,37,38]. However, even the expression of the partial inductances between single conductors in eccentric IMs is a highly complex one [39].

To overcome these difficulties, a novel method to calculate the inductance matrix of an eccentric IM is proposed in this paper by using WTA. Instead of directly deriving the partial inductance of a conductor in an IM with a non-uniform air gap, as in [39], this new proposal applies a conformal transformation [35,40] to obtain an equivalent, non-eccentric IM with a uniform air gap, which has much simpler inductance expressions. The problem is that this transformation also changes the angular positions of rotor conductors [41,42,43]. However, the flexibility of WTA makes it possible to deal with this non-uniform winding using routine tensor operations [30,44], which provide the final inductance matrix of the eccentric IM in a fast and very simple manner.

The structure of this work is as follows. In Section 2, the simple analytical model of the IM is presented, and in Section 3, the parameters of this model are presented for the case of a healthy machine. In Section 4, the novel approach for calculating the inductance matrix of an eccentric IM is theoretically presented. in Section 5, it is validated by comparing its results with the inductance matrix obtained with an FEM model, and in Section 6, it is applied to evaluate the degree a mixed eccentricity fault in two commercial IMs. Finally, Section 7 presents the conclusions of this work.

## 2. Simple Analytical Model of the IM

**Figure 1:**
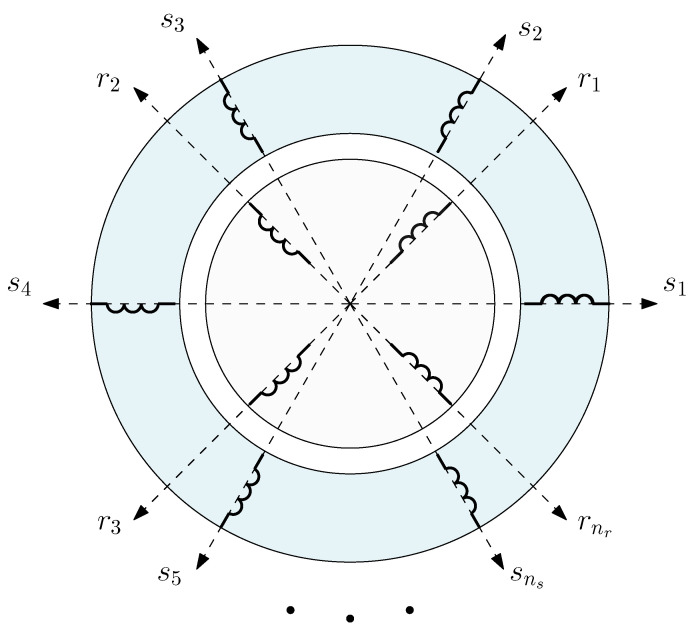
Reference frame of the IM with the axes of all stator windings ($s_{1},s_{2},\ldots ,s_{n_{s}}$) rigidly connected to the stator iron and conductors and those of the rotor windings ($r_{1},r_{2},\ldots ,r_{n_{r}}$) rigidly connected to the rotor iron and conductors.

The reference frame used to establish the analytical model of IM is a simple one, in which the reference axes of all stator windings are rigidly connected to the stator iron and conductors and those of the rotor windings are rigidly connected to rotor iron and conductors (the holonomic, Riemannian reference frame described in [45]). This reference frame is depicted in Figure 1 for a generic IM with $n_{s}$ stator windings and $n_{r}$ rotor windings, with a total number of windings $n=n_{s}+n_{r}$.

In this reference frame, the transient voltage equation along each axis has the simple form [30,46] for each of the machine windings

(1) $$e=Ri+\frac{d\varphi }{dt}$$

where *e* is the voltage applied to the winding, *R* is its resistance, and φ is the flux linkage of the winding. The *n* algebraic equations obtained by applying (1) to the *n* IM windings can be replaced by a single equation having the same form of (1) if each letter is replaced by the corresponding *n* matrix (first generalization postulate in [30]) as follows:

(2) $$e=Ri+\frac{d\varphi }{dt}$$

where

- $e={\left[e_{s_{1}},e_{s_{2}},\ldots ,e_{s_{n_{s}}},e_{r_{1}},e_{r_{2}},\ldots ,e_{r_{n_{r}}}\right]}^{t}$ is the voltage vector, which represents the terminal voltages applied to the *n* windings;
- $\varphi \;=\;{\left[{\varphi_{s}}_{1},{\varphi_{s}}_{2},\ldots ,{\varphi_{s}}_{n_{s}},{\varphi_{r}}_{1},{\varphi_{r}}_{2},\ldots ,{\varphi_{r}}_{n_{r}}\right]}^{t}$ is the flux linkage vector, which represents the flux linkages of the *n* windings;
- $i={\left[i_{s_{1}},i_{s_{2}},\ldots ,i_{s_{n_{s}}},i_{r_{1}},i_{r_{2}},\ldots ,i_{r_{n_{r}}}\right]}^{t}$ is the current vector, which represents the *n* winding currents;
- R is the resistance tensor. It is a square matrix, with $n^{2}$ components, for which its elements are winding resistances.

Moreover, ${}^{t}$ stands for the transpose operator. In addition, the relationship between the flux-linkage and the current vectors can be expressed as follows:

(3) φ=Li

where L is the inductance matrix, a square one, for which its $n^{2}$ components are the self-inductances and mutual inductances of the windings.

The torque equation in the reference frame of Figure 1 is given by the following [45]:

(4) $$T=J\frac{d\dot{\theta }}{dt}-\frac{1}{2}i^{t}\frac{dL}{d\theta }i$$

where *T* is the instantaneous applied shaft torque *T*, θ and $\dot{\theta }$ are the rotor instantaneous angle and speed, respectively, and *J* is the moment of inertia of the rotor.

It is worth mentioning that in (2) and (4), only the currents, voltages and torque at the IM terminals appear. That is, IM is considered as an analog of a closed box from which wires and shaft protrude [45]. This avoids the necessity of assuming that the current density and other waves are sinusoidally distributed in space, as in [46,47], which is advantageous because the eccentricity fault and the winding configuration generate harmonic spatial fields that distort their pure sinusoidal shape.

**Figure 2:**
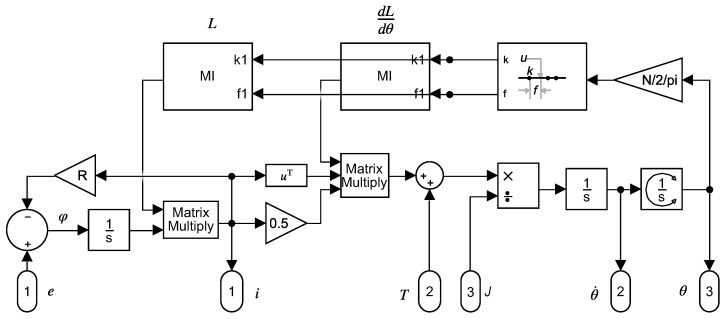
Dynamical model that implements (2) and (4) in Simulink. This model has three input ports: (1) the voltage vector e, (2) the applied shaft torque *T*, and (3) the moment of inertia of the rotor *J*. It has also three output ports: (1) the current vector i, (2) the rotor speed $\dot{\theta }$, and (3) the rotor angular position θ.

Figure 2 shows a Simulink model that implements both (2) and (4), using the model parameters R and L (and its angular derivative). The inductance matrix and its angular derivative depend on the rotor position and must be updated at each step of the simulation.

## 3. Determination of the Parameters of the IM Model

As stated in [48], the knowledge of the two sets of numbers R and L in (2)–(4) is sufficient to find the transient and steady-state performances of IM, assuming no magnetic saturation and no iron losses. These parameters can be found in the technical data provided by the manufacturer of the IM or calculated using its construction data (assuming healthy conditions), as in the case of the machine used for the experimental tests in this work. If these specifications are not available, they can be estimated using offline [49,50,51] or online parameter estimation techniques [52]. A comprehensive review of these techniques can be found in [53]. Recently, artificial intelligence (AI) methods have been proposed for parameter estimation in [54], as well as differential evolution algorithms [55]. Additionally, IM parameters change with temperature, frequency, or saturation, which has not been considered in the model used in with work.

**Figure 3:**
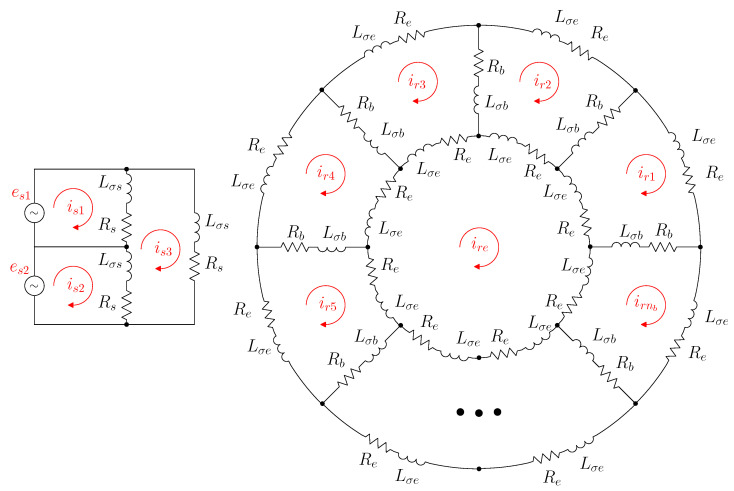
IM network. The three stator phases (**left**) have a delta connection, and each one has resistance $R_{s}$ and leakage inductance $L_{\sigma s}$. The rotor cage (**right**) has $n_{b}$ bars. Each rotor loop consists in two consecutive bars, each one with resistance $R_{b}$ and leakage inductance $L_{\sigma b}$. The bars are connected trough end ring segments, each one with resistance $R_{e}$ and leakage inductance $L_{\sigma e}$. The self and mutual inductances of the windings are not represented in this circuit.

The values of R and L that appear in (2) and (4) depend not only on the configuration of the IM windings but also on their connections. In this work (see Figure 3), the three stator phases have a delta connection. They are assumed to be identical, and each has a resistance $R_{s}$ and a leakage inductance $L_{\sigma s}$. The rotor cage has $n_{b}$ bars. Each rotor loop consists of two consecutive bars, each with a resistance $R_{b}$ and a leakage inductance $L_{\sigma b}$. The bars are connected via end ring segments, each with resistance $R_{e}$ and leakage inductance $L_{\sigma e}$. As in this work, the windings of the IM are considered to be in a healthy condition (only the eccentricity fault is addressed), and the resistances and leakage inductances of all the elements of the same type (stator phases, rotor bars, and rotor end ring segments) are considered to have the same values.

The assembly of R and L for the IM circuit represented in Figure 3 will not be performed directly, which needs a careful and cumbersome analysis of the circuit, especially for the calculation of the mutual inductance matrix. Instead, a much simpler approach is used in this paper by following Kron’s method [48]—that is, starting from a IM with the simplest connections, the primitive IM network, and using routine transformation rules for obtaining their final, complex values. As pointed out in [48], once matrices R and L are obtained for this simplest machine, the final values can be derived by an appropriate transformation that leaves the spatial position of all resistors and inductors undisturbed and changes only their interconnection, using basic tensor algebra.

**Figure 4:**
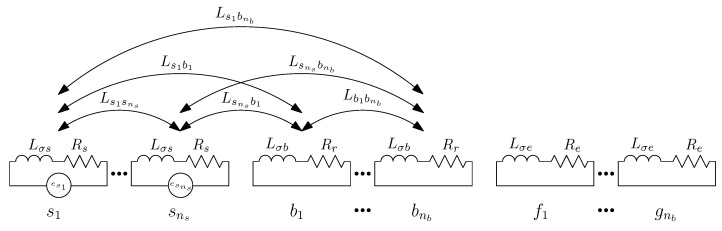
Primitive IM network, found by removing all interconnections between the windings and short circuiting each. The arrows show the mutual impedances between stator windings and cage bars. The end ring segments do not couple with the other windings through mutual impedances.

The simplest IM configuration that can be achieved without changing the spatial position of all resistors and inductors is obtained by removing all interconnections between the windings and short circuiting each [30], as shown schematically in Figure 4. In this “primitive” IM system [30], each stator phase, each of the $n_{b}$ cage bars, and each of the $2\;\cdot \;n_{b}$ end ring segments are considered as disconnected circuits, coupled only through mutual inductances, except for the end-ring segments, which do not have main flux linkages and only leakage flux ones. The resistance and inductance matrices of the IM in this primitive system ($R_{p}$ and $L_{p}$) are the simplest ones.

### 3.1. Resistance Matrix of the Primitive IM Network

The primitive IM network resistance matrix $R_{p}$ is diagonal, with the values corresponding to each stator phase, cage bar, and end ring segment along its diagonal. Since the IM windings are assumed to be in healthy conditions, the stator phases are considered to have the same resistance, $R_{s}$, the bar resistance $R_{b}$ is the same for all the bars, and the end ring segment resistance $R_{e}$ is the same for all end ring segments.

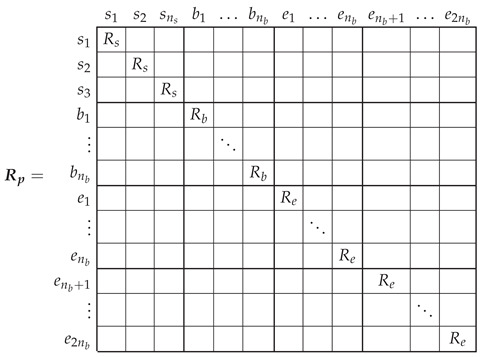
(5)

### 3.2. Inductance Matrix of the Primitive Network

The inductance matrix of the primitive IM network $L_{p}$ can be expressed as the sum of the inductance matrices corresponding to the main flux linkages, the main inductance matrix $L_{p\mu }$, and the leakage inductance matrix $L_{p\sigma }$, as follows.

(6) $$L_{p}=L_{p\mu }+L_{p\sigma }$$

The leakage inductance matrix $L_{p\sigma }$ elements are the inductances corresponding to end turns, end rings, and slots leakage, and they must be pre-calculated. This can be performed by using explicit expressions, such as those provided by [56,57,58], or obtained from the technical data provided by the manufacturer of the IM, as in this work. Only the analytical computation of $L_{p\mu }$ in (6) will be carried out in this work.

The leakage inductance matrix, $L_{p\sigma }$ in (6), is a diagonal matrix, with the values corresponding to each stator phase, cage bar, and end ring segment along its diagonal, as follows.

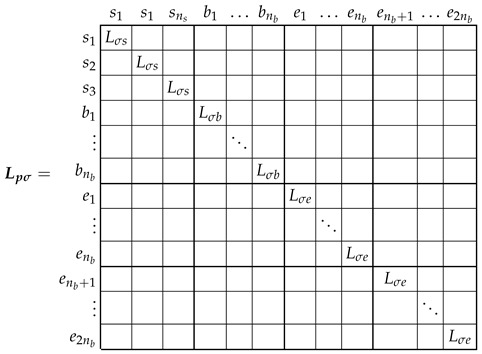
(7)

The main inductance matrix of the primitive IM network, $L_{p\mu }$ (6), has the following components.

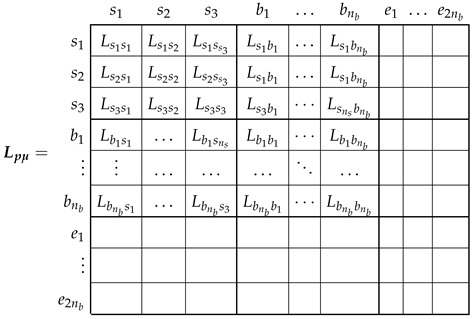
(8)

As displayed in (8), the mutual inductances between the end ring segments and the rest of the windings due to the main flux linkages are zero, because their only flux linkages are the leakage ones. As for the rest of the components of matrix $L_{p\mu }$, they depend on the actual stator and rotor winding configurations and on the angular position of the rotor. Among the many available methods in the technical literature for obtaining their values (FEM, WFA, etc.), the winding tensor approach has been selected in this work. It will be applied in the following section for the computation of matrix $L_{p\mu }$, both for the healthy and for the eccentric IM.

### 3.3. From the Primitive IM Network to the Actual One Using the Connection Matrix

**Figure 5:**
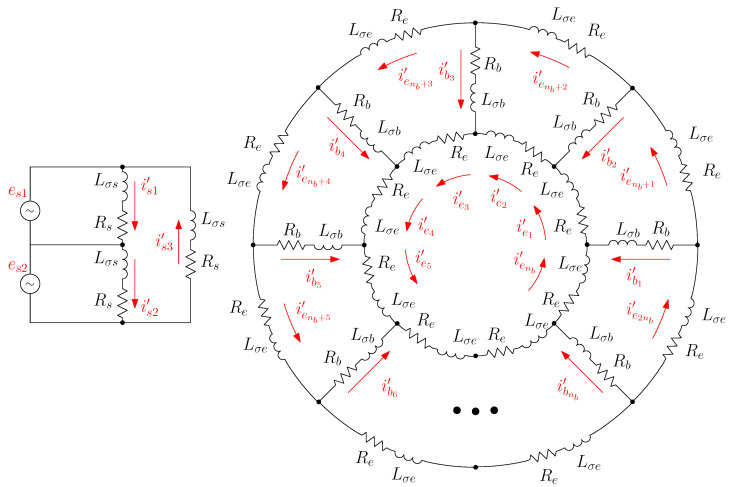
Interconnections of the elements the primitive IM network.

Once the $R_{p}$ and $L_{p}$ matrices of the primitive IM network (Figure 4) have been obtained, the R and L matrices of the actual IM network (Figure 3) can be obtained by simply specifying the interconnections of the elements of Figure 4, as depicted in Figure 5.

The currents flowing in each primitive network element, the branch currents $i^{\prime }$ in Figure 5, can be obtained from the loop currents, i in Figure 3, with the help of Kirchhoff’s laws, using transformation matrix $C_{p}$ as follows.

(9) $$i^{\prime }=C_{p}i$$

Therefore, the R and L matrices of the actual IM can be obtained from the $R_{p}$ (5) and $L_{p}$ (6) matrices of the primitive IM as follows [47].

(10) $$R=C_{p}^{t}R_{p}C_{p}$$

(11) $$L=C_{p}^{t}L_{p}C_{p}$$

The transformation matrix $C_{p}$ only reflects the interconnections between the primitive elements and contains only zeros, ones, and minus ones. It is given, by direct comparison between Figure 3 and Figure 5, as follows.

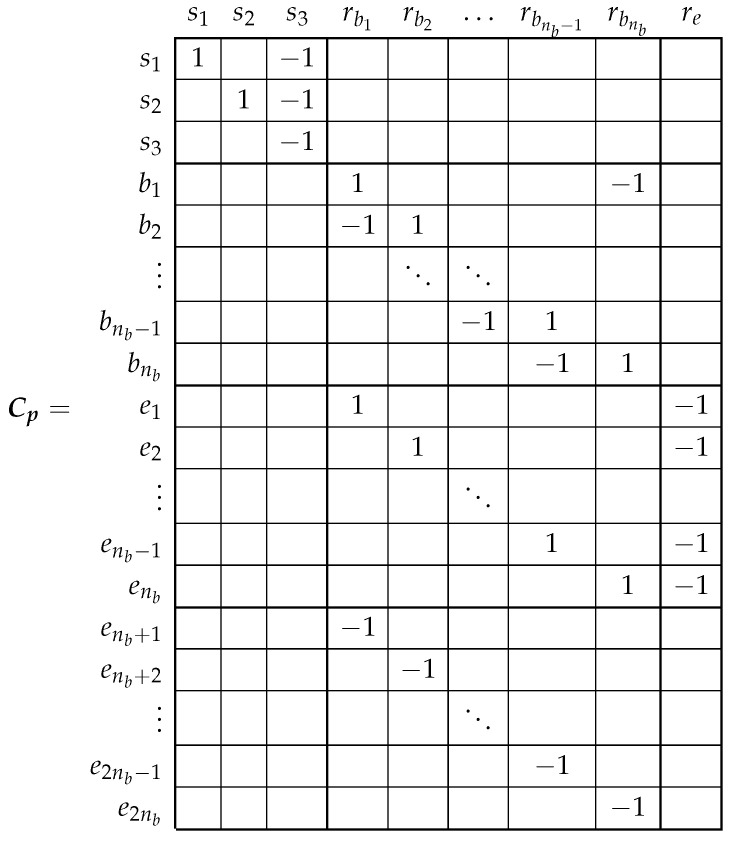
(12)

It is worth mentioning that the set of loop currents used in Figure 3 is not unique. For example, in [36,38], another set of rotor loops currents has been used, with $n_{b}-1$ rotor loops containing a rotor bar and two loops for the currents in the rotor end-rings segments. As [47] states, they are simply different expressions of the current tensor, providing the same values for the actual bar and end-ring segment currents. Another advantage of using (10) and (11) is that transformation matrix $C_{p}$ can be used to represent not only the IM network under healthy conditions but also under faulty conditions, such as multiple bar and end-ring breakages, as in [38]. This extends the application of the model shown in Figure 2 to the field of multiple fault analysis.

## 4. Computation of the Main Inductance Matrix of the Healthy and the Eccentric IM Using the Conformal Winding Tensor Approach

In this section, the main inductance matrix $L_{p\mu }$ (8) of the primitive IM network is calculated using the winding tensor approach. If iron saturation and losses are neglected, as in the present work, mutual inductances depend only on the geometry of the system [59]. Other assumptions are that the iron permeability is infinite and that only the radial component of the main flux that crosses the smooth air gap is considered in this work. The calculation of the mutual inductances considering also the tangential component of the flux can be found in [37,60]. A higher precision can be achieved using numerical methods, such as those based in FEM [22,23], but at the cost of an increased computational complexity. However, the simple, analytical approach followed in this work has proven to be able to correctly reproduce the fault harmonics of the mixed eccentricity fault, with a low computational load.

The methodology proposed in this work follows the same approach as in the previous section, using a single conductor as the most primitive component of any winding:

1. A primitive spatial network, similarly to Figure 4, is constructed by removing all interconnections between winding conductors and short circuiting each one without changing their spatial positions. For this simple network, the matrix with the partial inductances between conductors is obtained, which makes it easier to take into account the effect of IM eccentricity.
2. A transformation matrix, similarly to (12), is constructed. It represents the interconnections of the conductors of each winding for each angular position of the rotor, i.e., the winding tensor.
3. The main inductance matrix of the primitive IM network (8) is obtained from the partial inductance matrix of the conductors using a routine tensor transformation with the winding tensor, similarly to (11).

In order to represent any winding spatial distribution using the interconnection of elemental conductors, the circular air gap is equally divided into *N* segments, and each of them is filled with an elementary conductor located in the air gap zone. In [37], two layers of conductors have been considered instead: one placed on the inner stator surface and the other one placed on the outer rotor surface, which allows considering the effects of the tangential flux in the air gap. However, considering that the air gap length is small and that the focus of the paper is to introduce the use of the conformal transformation applied to the winding tensor, the simplest approach that considers only one layer of conductors has been followed. The maximum number of spatial harmonics of the winding that can be represented using these elementary conductors is N/2. Therefore, a high value of *N* has been chosen, N=3600, as in [36].

**Figure 6:**
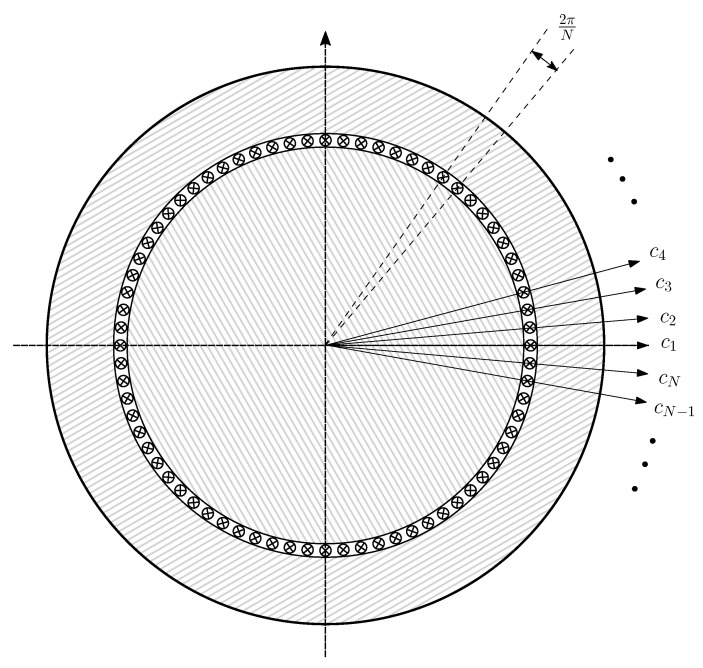
Elementary conductors placed in the air gap that constitute the primitive spatial network of the IM. These *N* conductors are considered to be disconnected, and their currents are considered to be independent variables.

The N×N matrix that contains the partial inductances between the conductors of Figure 6, $L_{c\mu }$, is given by the following: 
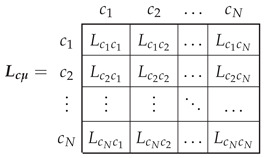
(13)
for which component (m,n), $L_{c_{m}c_{n}}$, is the mutual partial inductance [37] between the conductors placed at positions $\left(m-1\right)\;\cdot \;\frac{2\pi }{N}$ and $\left(n-1\right)\;\cdot \;\frac{2\pi }{N}$, with m,n=1,2,…,N.

In the case of an IM with uniform air gap, as shown in Figure 6, and considering that the air gap is small compared to its radius, the components $L_{c_{m}c_{n}}$ of (13) depend solely on the angular separation between conductors *m* and *n*, and they are given by the following [61]:

(14) $$L_{c\mu }\left(m,n\right)=L_{c_{m}c_{n}}=\frac{\mu_{0}\ell r\pi }{g}\;\cdot \;{\left(\frac{1}{2}-\frac{\left|m-n\right|}{N}\right)}^{2}$$

where $\mu_{0}=4\pi 10^{-7}$H/m, *ℓ* is the effective length of the stator bore, *r* is the radius at the centre of the air gap, and *g* is the length of the air gap.

The relationship between the currents in the winding conductors $i_{c}$ (Figure 6) and the currents in the primitive IM network $i^{\prime }$ (Figure 5) can be formulated using a $\left(N\times \left(n_{s}+3n_{b}\right)\right)$ connection matrix and the winding tensor $C_{c}$ [36,38] as follows:

(15) $$i_{c}=C_{c}\;\cdot \;i^{\prime }$$

where the following is the case.

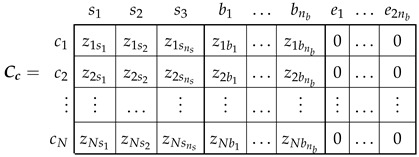
(16)

The connection matrix $C_{c}$ indicates the connections between the conductors of each winding. Its (i,j) element contains the number of conductors $z_{ij}$ of winding *j* contained in the angular interval of length 2π/N (Figure 6), centered at $\left(i-1\right)\;\cdot \;\frac{2\pi }{N}$, with the corresponding sign depending on the direction of the current. Since the rotor end rings do not have any conductors in the air gap, the corresponding columns in $C_{c}$ are zero. These columns have been maintained in (16) for the sake of completeness.

The main inductance matrix of the windings of Figure 5, $L_{p\mu }$ (8) is obtained from the partial inductance matrix of the conductors $L_{c\mu }$ (13) using the connection matrix (16) as follows.

(17) $$L_{p\mu }={C_{c}}^{t}\;\cdot \;L_{c\mu }\;\cdot \;C_{c}$$

The winding connection matrix $C_{c}$ (16) must be obtained for the *N* possible angular positions of the rotor ($\theta_{k}=\left(k-1\right)\;\cdot \;\frac{2\pi }{N}$, with k=1,…,N). However, the columns of $C_{c}$ corresponding to the $n_{s}$ stator windings do not depend on the rotor position, and the columns of $C_{c}$ corresponding to the rotor windings for a given rotor position $\theta_{k}$ are the same as the columns defined with the rotor at the origin ($\theta_{0}=0$), but circularly shifted *k* positions.

In (16), no restrictions are imposed on the connections of the conductors of each winding, which can be arbitrarily complex, as in the case of asymmetrical windings (turn-to-turn short circuits, etc.). However, in the case of a machine with a healthy winding configuration, the conductor distributions in all stator and rotor windings are the same, respectively. Therefore, the column of $C_{c}$ corresponding to the *k*th stator winding ($s_{k}$) is equal to the column of the first stator winding ($s_{1}$) but circularly shifted $k\;\cdot \;N/n_{s}$ positions. The same applies to the rotor windings, but in this case, the circular shift is $k\;\cdot \;N/n_{b}$ positions. In this particular case and based on the circulant properties of matrix $L_{c\mu }$, the calculation of (16) can be performed very quickly with the convolution theorem, using the fast Fourier transform (FFT), as presented in [61].

### 4.1. Partial Inductance Matrix of the Conductors in an Eccentric IM

**Figure 7:**
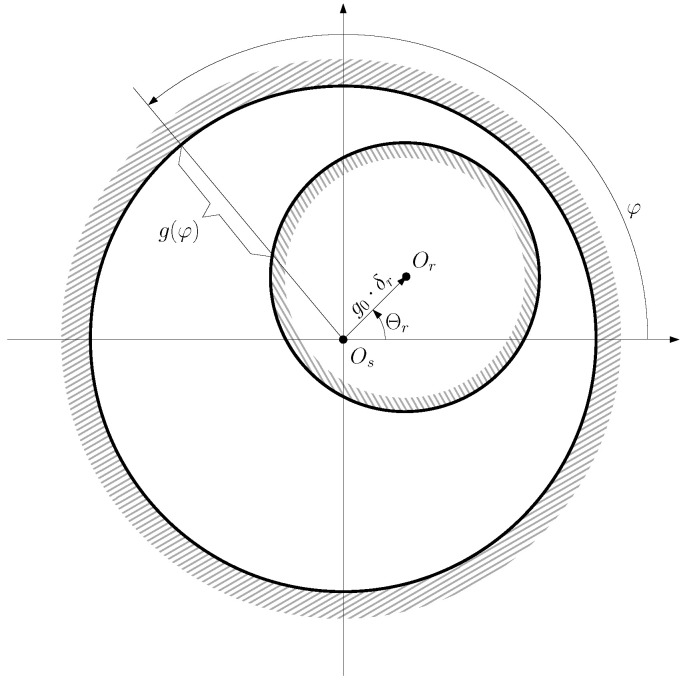
Air gap length g(φ) of an eccentric machine as a function of the angular coordinate φ, which depends on the position of the rotor centre $O_{r}$ with respect to the stator centre $O_{s}$.

In cases rotor eccentricity, the rotor center $O_{r}$ does not coincide with stator centre $O_{s}$ (Figure 7), which results in a non-uniform air gap length that invalidates (14).

**Figure 8:**
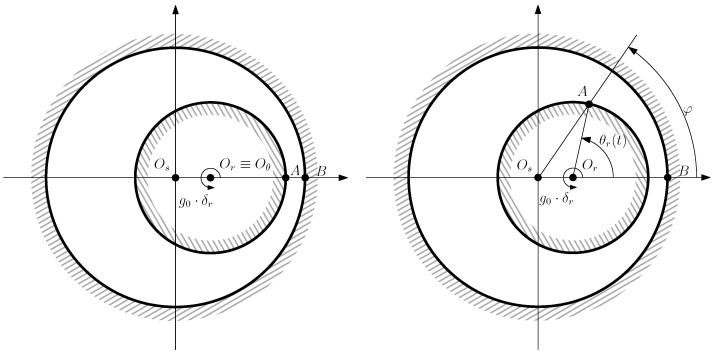
Pure static eccentricity. Relative position of a rotor conductor, *A*, and a stator conductor, *B*, when the rotor turns an angle $\theta_{r}\left(t\right)$ (**right**) from the initial line (**left**), in the case of SE. The minimum air gap length is always located at the position of the stator conductor *B*.

**Figure 9:**
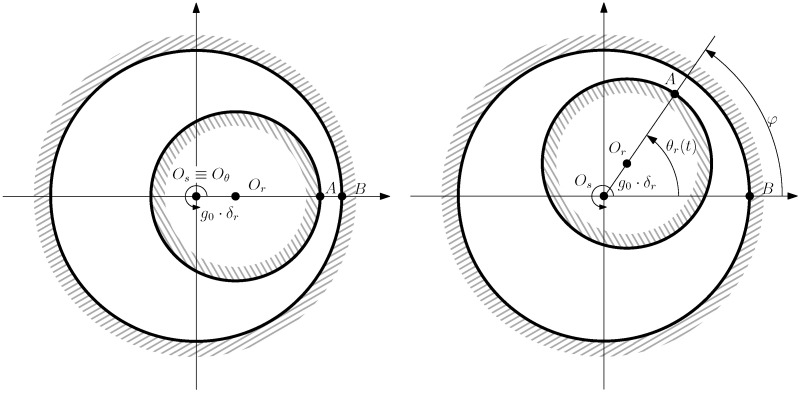
Pure dynamic eccentricity. Relative position of a rotor conductor, *A*, and a stator conductor, *B*, when the rotor turns an angle $\theta_{r}\left(t\right)$ (**right**) from the initial line (**left**), in the case of DE. The minimum air-gap length is always located at the position of the rotor conductor *A*.

**Figure 10:**
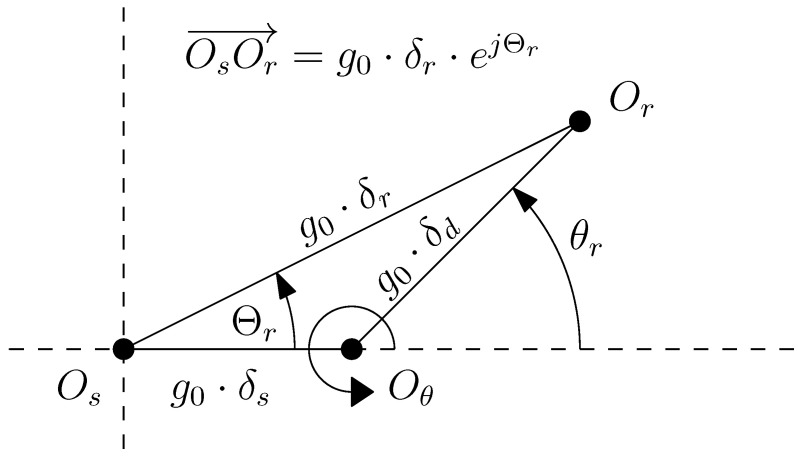
Position of the rotor centre ($O_{r}$), the stator centre ($O_{s}$), and the axis of rotation ($O_{\theta }$) in case of an IM witth ME eccentricity ($\delta_{r}$), as a geometric combination of static ($\delta_{s}$) and dynamic ($\delta_{d}$) eccentricity. $\theta_{r}$ represents the angle of rotation of the rotor.

From Figure 7, the position of the rotor centre can be represented using its radial coordinate, $\delta_{r}\;\cdot \;g_{0}$, and its angular coordinate $\Theta_{r}$ follows:

(18) $$\vec{O_{s}O_{r}}=g_{0}\;\cdot \;\delta_{r}\;\cdot \;e^{j\Theta_{r}}\;0\leq \delta_{r}<1,\;0\leq \Theta_{r}<2\pi$$

where $g_{0}$ is the air gap length of the IM without any eccentricity, and $\delta_{r}$ is the degree of eccentricity ($0\leq \delta_{r}<1$). The type of eccentricity, as well as the degree of eccentricity $\delta_{r}$, depends on the position of the axis of rotation of the rotor ($O_{\theta }$) as follows:

- A pure static eccentricity (SE) is characterized (Figure 8) by a displacement of the axis of rotation of the rotor ($O_{\theta }$) with respect to the geometric center of the stator ($O_{s}$). The axis of rotation of rotor $O_{\theta }$ coincides with the geometric center of the rotor. It can be caused by misalignments of the mounted bearings or of the bearing plates. The rotor is not centered with the stator bore, but it rotates around its own geometric centre: that is, $\Theta_{r}$ = constant in Figure 7. The air gap length is non uniform, but its shape does not change when the rotor turns (Figure 8).

- A pure dynamic eccentricity (DE) is characterized (Figure 9) by a displacement of the geometric centre of the rotor ($O_{r}$) from its axis of rotation ($O_{\theta }$), which coincides with the axis of the stator bore ($O_{s}$). It can be caused by a manufacturing defect, a bent shaft, bearings defects, etc. Under DE, the center of the rotor rotates along a circular path in Figure 7, with the same speed as the rotor. In this case, the position of the minimum air gap rotates with the rotor (Figure 9)
- A mixed eccentricity fault (ME) consists of the simultaneous presence of SE and DE (Figure 10). In this case, the axis of rotation ($O_{\theta }$ in Figure 10) is displaced both from the geometric center of the stator ($O_{s}$), as in the case of pure static eccentricity, and from the centre of the rotor ($O_{r}$), as in the case of pure dynamic eccentricity.

From Figure 10, the coordinates ($g_{0}\;\cdot \;\delta_{r}$ and $\Theta_{r}$) of the rotor center depend on the angular position of the rotor $\theta_{r}$ and the degree of static $\delta_{s}$ and dynamic $\delta_{d}$ eccentricity of the machine (see Figure 10) as follows.

(19) $$\Theta_{r}\left(\theta_{r}\right)=\tan^{-1}\left(\frac{\delta_{d}\sin\left(\theta_{r}\right)}{\delta_{s}+\delta_{d}\cos\left(\theta_{r}\right)}\right),\;\;\;\;\;\delta_{r}\left(\theta_{r}\right)=\sqrt{{\delta_{s}}^{2}+{\delta_{d}}^{2}+2\delta_{s}\delta_{d}\cos\left(\theta_{r}\right)}$$

Each component (*m* and *n*) of the induction matrix $L_{cm_{mn}}$ in an eccentric IM depends not only on the angular separation between conductors *m* and *n* but also on their absolute position and on the position of the center of the rotor, for which its coordinates ($g_{0}\;\cdot \;\delta_{r}$, $\Theta_{r}$) are, in turn, functions of the angular position of the rotor (19). The corrected value of the partial inductance between conductors that replaces (14) in an eccentric IM has been given in [36,39]. In the general case of a machine with ME, the inverse of the air gap length is a function of the coordinates of the rotor centre (18) given by the following [23]:

(20) $$g{\left(\varphi ,\Theta_{r},\delta_{r}\right)}^{-1}=g_{0}^{-1}\;\cdot \;\left(A_{0}+\sum_{m=1}^{n_{t}}A_{m}\;\cdot \;\cos\left(m(\varphi -\Theta_{r})\right)\right)$$

where the following is the case:

(21) $$A_{0}=\frac{1}{\sqrt{1-{\delta_{r}}^{2}}}\;\;\;\;\;\;\;\;\;\;\;\;\;\;\;A_{m}=2{\left(\frac{1-\sqrt{1-{\delta_{r}}^{2}}}{\sqrt{1-{\delta_{r}}^{2}}}\right)}^{m}\;m=1\ldots n_{t}$$

and the number $n_{t}$ of terms can be chosen to achieve the desired precision (one term in [62,63,64,65] and two terms in [32]). Using (20), the expression that replaces (14) for a given rotor position $\theta_{k}=\left(k-1\right)\;\cdot \;\frac{2\pi }{N}$, with k=1,2,…,N, is given by the following [37]:

(22) $$L_{c\mu }\left(m,n\right){|}_{k}=\frac{\mu_{0}lr}{g_{0}}\;\cdot \;\Lambda \left(m\frac{2\pi }{N},n\frac{2\pi }{N},\Theta_{r}\left(k\frac{2\pi }{N}\right),\delta_{r}\left(k\frac{2\pi }{N}\right)\right)$$

where the following is the case:

(23) $$\begin{array}{c} \Lambda \left(\alpha ,\varphi ,\Theta_{r},\delta_{r}\right)=\frac{A_{0}}{4\pi }{\left(\varphi -\alpha \right)}^{2}+\sum_{m=1}^{n_{t}}\frac{A_{m}}{2\pi }\left(\frac{\left(\varphi -\alpha \right)\sin\left(m(\varphi -\Theta_{r})\right)}{m}+\frac{\cos\left(m(\varphi -\Theta_{r})\right)}{m^{2}}\right)-\\ \left(\frac{1}{2}-K\left(\alpha ,\Theta_{r},\delta_{r}\right)\right)\;\cdot \;\left(A_{0}\left(\varphi -\alpha \right)+\sum_{m=1}^{n_{t}}A_{m}\frac{\sin\left(m(\varphi -\Theta_{r})\right)}{m}\right) \end{array}$$

and the following is obtained.

(24) $$K\left(\alpha ,\Theta_{r},\delta_{r}\right)=\sum_{m=1}^{n_{t}}\frac{A_{m}}{2\pi A_{0}}\frac{\sin(m\left(\Theta_{r}-\alpha \right))}{m}$$

### 4.2. Simplified Formulation of the Partial Inductance between Conductors in Case of Rotor Eccentricity with the Conformal Winding Tensor Approach

Although (22) provides a closed analytical expression of the partial inductance between conductors in an eccentric IM, it is much more complex than (13), which makes it difficult to implement, especially in small devices for on-line fault diagnosis. In contrast, with the method proposed in this work, the simplicity of (13) is retained, even in case of a high degree of rotor eccentricity.

The main idea behind the approach proposed in this work is to transform the non-uniform air gap into a uniform one using a conformal transformation, the Moebius transformation, so that the simple expression given by (14) can be used in this transformed domain. As the conformal transformation preserves the electromagnetic energy of the windings, the mutual inductances between any two windings are preserved. Therefore, the values of the winding inductances obtained in this simple domain are the same than in the original eccentric domain.

In [42,43], it has been shown that the Moebius transformation can be applied to convert this eccentric IM into a non-eccentric machine with uniform air gap length. It is given by the following:

(25) $$w\left(z\right)=\frac{\hat{a}z+\hat{b}}{\hat{c}z+1}$$

where *z* is the coordinate of a point in the air-gap of the eccentric machine, and *w* is the coordinate of the same point in the air-gap of the non-eccentric machine generated by the conformal transformation (25). The factors $\hat{a}$, $\hat{b}$, and $\hat{c}$ depend only on the geometrical characteristics of IM and on the degree of eccentricity, and their expressions are given in this section.

**Figure 11:**
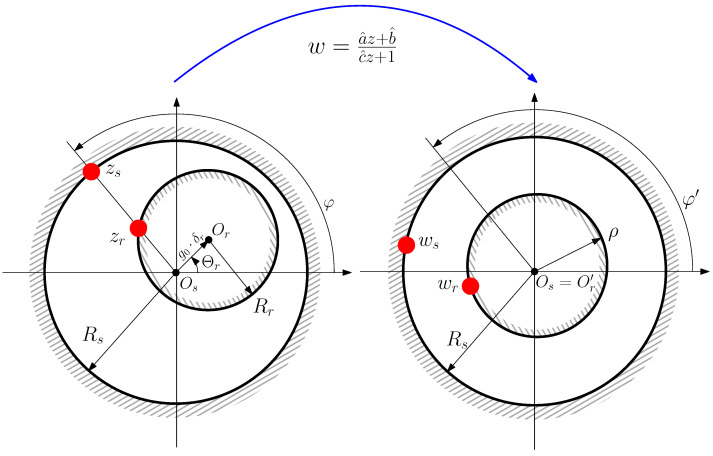
The Moebius transformation of an eccentric IM with non-uniform air gap (**left**) gives a non-eccentric IM with a uniform air gap (**right**), but with modified conductor angular positions and with a different rotor radius.

The result of applying transformation (25) to the eccentric machine is a non-eccentric machine, with concentric rotor and stator surfaces, as seen in see Figure 11. However, in the transformed machine, the length of the rotor radius changes and the positions of the conductors are at different angular positions than the original ones.

The radius of the outer surface of the rotor ρ of the transformed IM is given by the following (see Figure 11)

(26) $$\rho =\frac{R_{s}^{2}+R_{r}^{2}-{\left(g_{0}\delta_{r}\right)}^{2}-\sqrt{{\left(R_{s}^{2}+R_{r}^{2}-{\left(g_{0}\delta_{r}\right)}^{2}\right)}^{2}-4R_{s}^{2}R_{r}^{2}}}{2R_{r}}$$

where $R_{s}$ is the radius of the inner stator surface, and $R_{r}$ is the radius of the outer rotor surface. Therefore, the air gap of the transformed IM has a uniform length of the following:

(27) $$g^{\prime }=R_{s}-\rho$$

and a mean radius equal to the following.

(28) $$r^{\prime }=\frac{R_{s}+\rho }{2}$$

The coefficients of (25) that perform the transformation from the eccentric machine into the non-eccentric machine of Figure 11 are the following ones.

(29) $$\hat{a}=e^{\Theta_{r}}$$

(30) $$\hat{b}=\frac{R_{s}^{2}\left(\rho -R_{r}+g_{0}\delta_{r}\right)}{R_{s}^{2}-R_{r}\rho +\rho g_{0}\delta_{r}}$$

(31) $$\hat{c}=\frac{\rho -R_{r}+g_{0}\delta_{r}}{R_{s}^{2}-R_{r}\rho +\rho g_{0}\delta_{r}}e^{\Theta_{r}}$$

**Figure 12:**
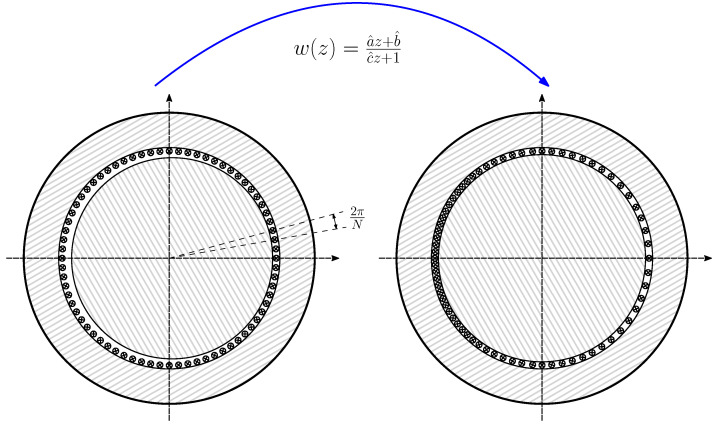
Moebius transformation of the elementary conductors placed in the air gap that constitute the primitive spatial network of the IM, given in Figure 6. The original set of *N* equally spaced elementary conductors placed in the non-uniform air gap of the eccentric IM (**left**), becomes a set of *N* elementary conductors with non-uniform spacing in the smooth air gap of the transformed IM (**right**).

However, the transformed machine has concentric rotor and stator surfaces, but the conductors are at different angular positions than in the original, eccentric IM. In this manner, the reference frame of Figure 6, which consists of *N* equally spaced elementary conductors placed in the air gap, is transformed into a set of *N* elementary conductors with non-uniform conductors spacing, as shown in Figure 12.

For a given rotor position $\theta_{k}=\left(k-1\right)\;\cdot \;\frac{2\pi }{N}$ (with k=1,2,…,N), the expression of the mutual inductance between two elementary conductors m,n (with m,n=1,2,…,N), placed in the air gap of the eccentric IM at positions $z_{m}=r\;\cdot \;\exp\left(j\left(m-1\right)2\pi /N\right)$ and $z_{n}=r\;\cdot \;\exp\left(j\left(n-1\right)2\pi /N\right)$, can be now easily obtained in the transformed IM, using (25), (27), and (28), as follows:

(32) $$L_{c\mu }\left(m,n\right){|}_{k}=\frac{\mu_{0}\ell r^{\prime }\pi }{g^{\prime }}\;\cdot \;{\left(\frac{1}{2}-\frac{\left|\mathrm{angle}\left(w(re^{j\left(m-1\right)\frac{2\pi }{N}})\right)-\mathrm{angle}\left(w(re^{j\left(n-1\right)\frac{2\pi }{N}})\right)\right|}{2\pi }\right)}^{2}$$

where $r=(R_{s}+R_{r})/2$. The expression (32) replaces (14) for the case of an eccentric IM and is much simpler to apply than (22). It is worth mentioning that although the rotor position $\theta_{k}$ does not appear explicitly in (32), it does affect the calculation of parameters ρ (26), $\hat{a}$ (29), $\hat{b}$ (30), and $\hat{c}$ (31).

## 5. Numerical Validation

**Figure 13:**
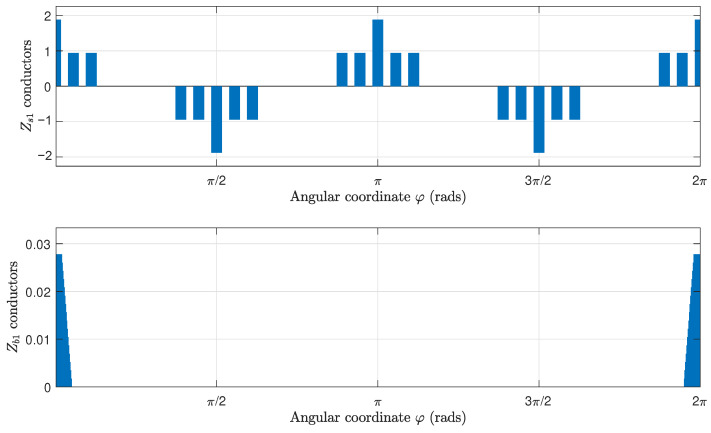
Number and direction of the conductors per air gap interval of a stator winding (**top**) and a rotor bar (**bottom**).

**Figure 14:**
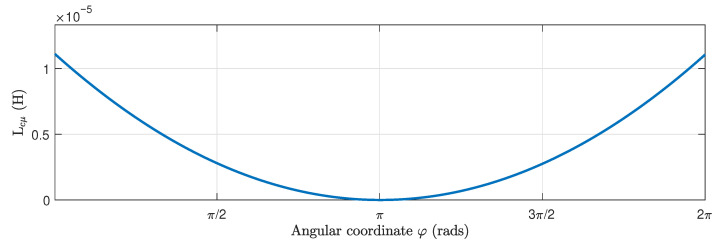
Mutual inductance between an elementary conductor placed at the origin and an elementary conductor placed at a given angular coordinate φ for the IM given in Appendix A, without eccentricity. This corresponds to the first column of matrix $L_{c\mu }$ (13).

In this section, the proposed method is applied to an industrial IM, for which its data are given in Appendix A. Figure 13 shows the components of the winding tensor $C_{c}$ (16), which contains the distribution of the conductors of the stator windings and the rotor bars, for each rotor position. They corresponds to the first and the fourth columns, respectively, of matrix $C_{c}$ (16). Figure 14 shows the mutual inductance between an elementary conductor placed at the origin and an elementary conductor placed at a given angular coordinate φ for the same IM, without eccentricity. This corresponds to the first column of matrix $L_{c\mu }$ (13).

The stator winding has three phases, and the rotor winding of this motor is made up of 28 rotor loops, giving a total of 31 IM phases. This motor has been modeled using a 2D finite elements approach, with the open source software FEMM, and 1000 equally spaced rotor angular positions have been used for the simulations. The skew of rotor bars has been taken into account using a multi-slice approach. Three different degrees of static eccentricity, $\delta_{s}=\left[0.2,0.4,0.6\right]$, and dynamic eccentricity, $\delta_{d}=\left[0.2,0.4,0.6\right]$, as well as their possible combinations (mixed eccentricity) have been analyzed. In addition, the case of healthy machine ($\delta_{s}$ = 0, $\delta_{d}$ = 0) has been considered for comparison purposes. This produces a total number of 10 degrees of eccentricity: ($\delta_{s}$ = 0, $\delta_{d}$ = 0), ($\delta_{s}$ = 0, $\delta_{d}$ = 0.2), ($\delta_{s}$ = 0, $\delta_{d}$ = 0.4), ($\delta_{s}$ = 0, $\delta_{d}$ = 0.6), ($\delta_{s}$ = 0.2, $\delta_{d}$ = 0), ($\delta_{s}$ = 0.2, $\delta_{d}$ = 0.2), ($\delta_{s}$ = 0.2, $\delta_{d}$ = 0.4), ($\delta_{s}$ = 0.4, $\delta_{d}$ = 0), ($\delta_{s}$ = 0.4, $\delta_{d}$ = 0.2), and ($\delta_{s}$ = 0.6, $\delta_{d}$ = 0).

**Figure 15:**
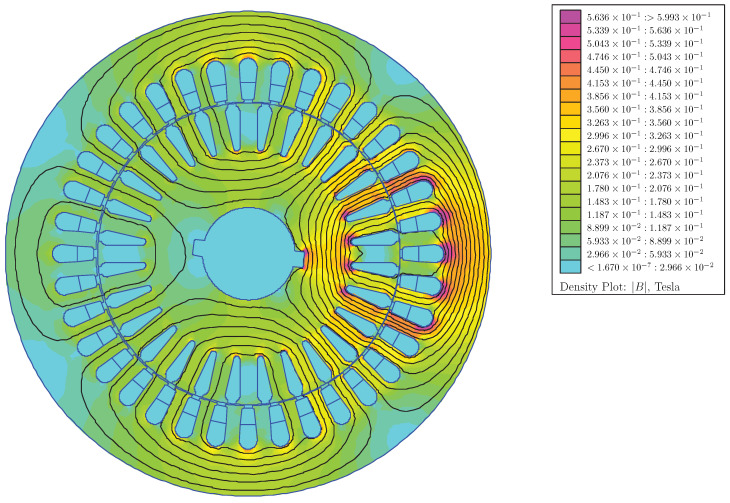
FEM simulation of the IM of Appendix A for a mixed eccentricity with a static eccentricity degree of 40% ($\delta_{s}$ = 0.4) and a dynamic eccentricity degree of 20% ($\delta_{d}$ = 0.2), with only the first stator phase fed with a 1 A constant current.

**Figure 16:**
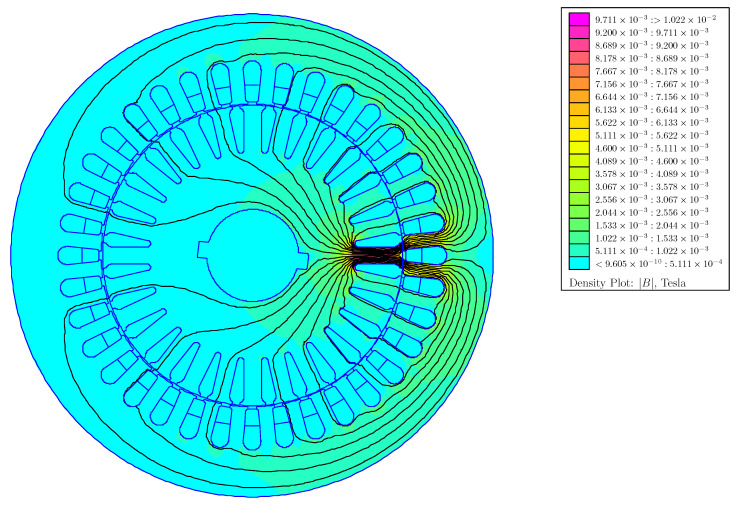
FEM simulation of the IM of Appendix A for a mixed eccentricity with a static eccentricity degree of 40% ($\delta_{s}$ = 0.4) and a dynamic eccentricity degree of 20% ($\delta_{d}$ = 0.2), with only the first rotor phase fed with a 1 A constant current.

The mutual and self inductances between all stator and rotor phases have been obtained with the following method: for each of the 10 combinations of static and dynamic rotor eccentricity and for each rotor angular position (1000), each one of the IM phases is fed with a 1 A constant current, and the flux linkages of all IM phases are evaluated, which gives all mutual inductances and the self-inductance of the fed phase for that rotor position and type and degree of eccentricity. Figure 15 shows the FEM simulation for the first stator phase, and Figure 16 shows the FEM simulation for the first rotor phase, both for a mixed eccentricity with a static eccentricity degree of 40% ($\delta_{s}$ = 0.4) and a dynamic eccentricity degree of 20% ($\delta_{d}$ = 0.2).

This process must be repeated for the 31 IM phases, which gives a total number of $31\times 10^{4}$ FEM simulations. Each simulation takes an average wall time of 30 s on the computer platform of Appendix B, which represents a total wall time of 2583 h to complete the construction of the IM inductance matrix for the 10 eccentricity degrees considered in this work.

On the contrary, the construction of the IM inductance matrix for the 10 eccentricity degrees using the method proposed in this work, the conformal winding tensor approach, requires only 700 s of wall time to complete on the same computing platform, which is only 0.45% of the time that FEA needs. This speed can be a decisive edge when analyzing, for example, the case of axial eccentricity, which results in a continuous variation of the degree of eccentricity along the shaft and requires much more than the 10 cases analyzed in this work to be accurately reproduced by the IM model.

It is worth mentioning that the inductance matrix of this eccentric IM has also been obtained using the method described in [39], and it is compared with the results presented in this work. The inductance matrix obtained with both methods is the same, up to machine precision, because they are based on the same analytical equations. Nevertheless, the expressions of the partial inductance of a single conductor in an eccentric IM (19)–(24) are much more complex than the simple one used in this work (32), and the time needed to solve them (3300 s) is also much longer. Finally, the method presented in [39] does not make use of the winding tensor, which makes it difficult to apply it to the analysis of multiple and simultaneous IM faults.

**Figure 17:**
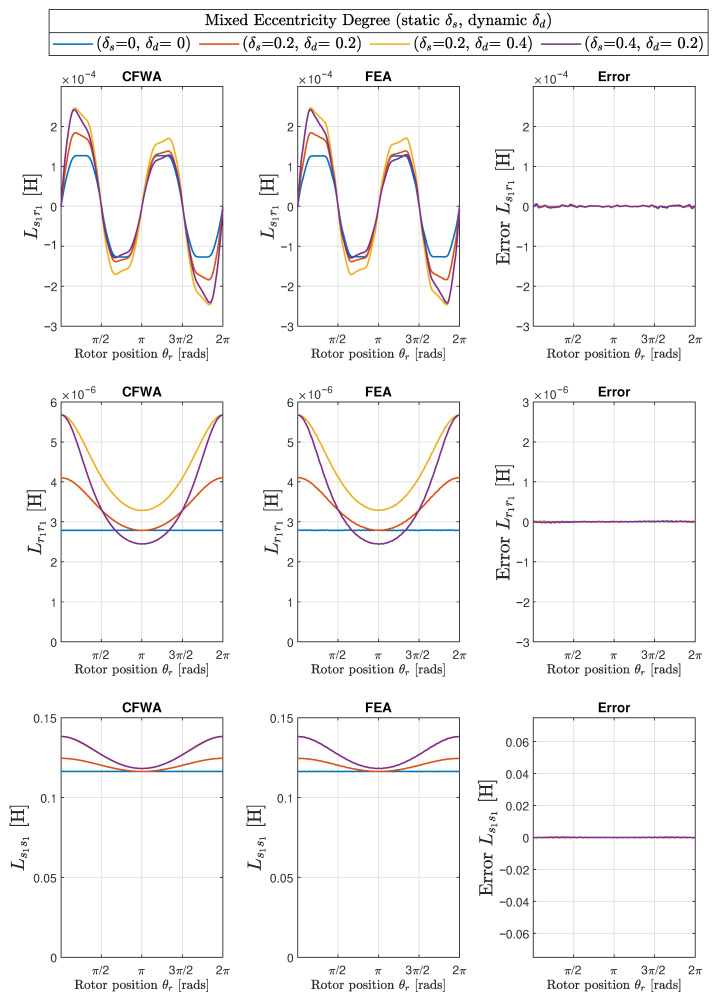
Mutual inductance between the first stator and the first rotor phase ($L_{s_{1}r_{1}}$, **top row**) and self-inductances of the first rotor phase ($L_{r_{1}r_{1}}$, **middle row**) and of the first stator phase ($L_{s_{1}s_{1}}$, **bottom row**), for the IM of Appendix A, with three different degrees of static ($\delta_{s}$) and dynamic ($\delta_{d}$) eccentricity: ( $\delta_{s}$ = 0.2, $\delta_{d}$ = 0.2), ($\delta_{s}$ = 0.2, $\delta_{d}$ = 0.4), and ($\delta_{s}$ = 0.4, $\delta_{d}$ = 0.2). The case of healthy machine ($\delta_{s}$ = 0.0, $\delta_{d}$ = 0.0) has also been included for comparative purposes. The first column presents the results obtained with the conformal winding tensor approach, the second column contains the results obtained with FEA, and the third column contains the errors between both approaches.

**Figure 18:**
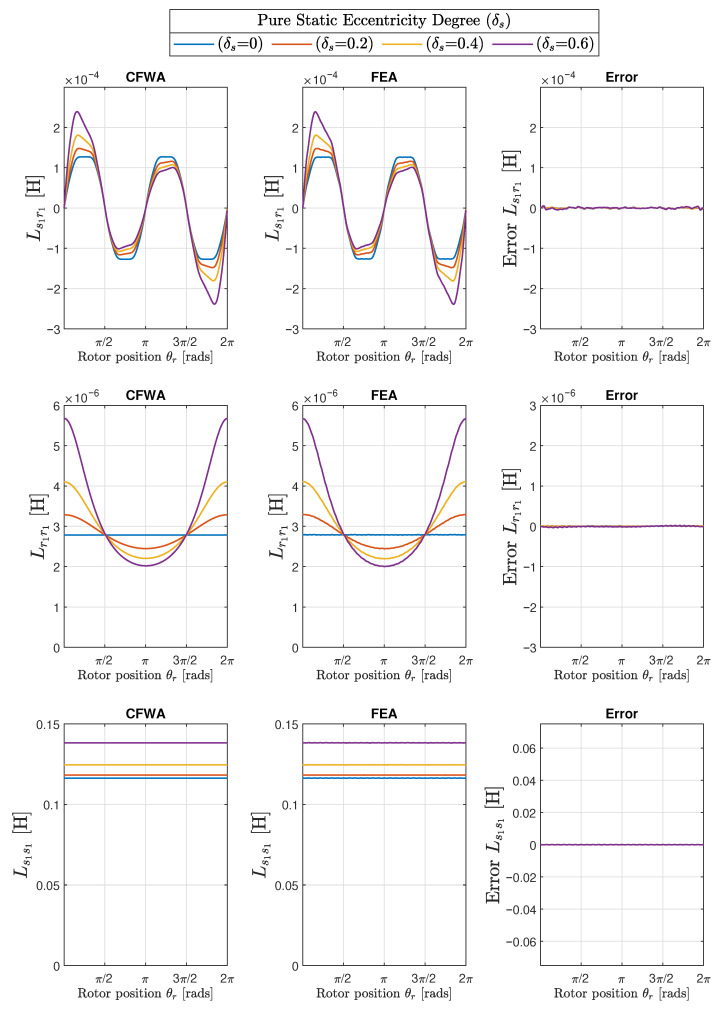
Mutual inductance between the first stator and the first rotor phase ($L_{s_{1}r_{1}}$, **top row**) and self-inductances of the first rotor phase ($L_{r_{1}r_{1}}$, **middle row**) and of the first stator phase ($L_{r_{1}r_{1}}$, **bottom row**) for the IM of Appendix A, with three different degrees of static ($\delta_{s}$) eccentricity: $\delta_{s}$ = 0.2, $\delta_{s}$ = 0.4, and $\delta_{s}$ = 0.6. The case of healthy machine $\delta_{s}$ = 0.0 has also been included for comparative purposes. The first column presents the results obtained with the conformal winding tensor approach, the second column presents the results obtained with FEA, and the third column presents the errors between both approaches.

**Figure 19:**
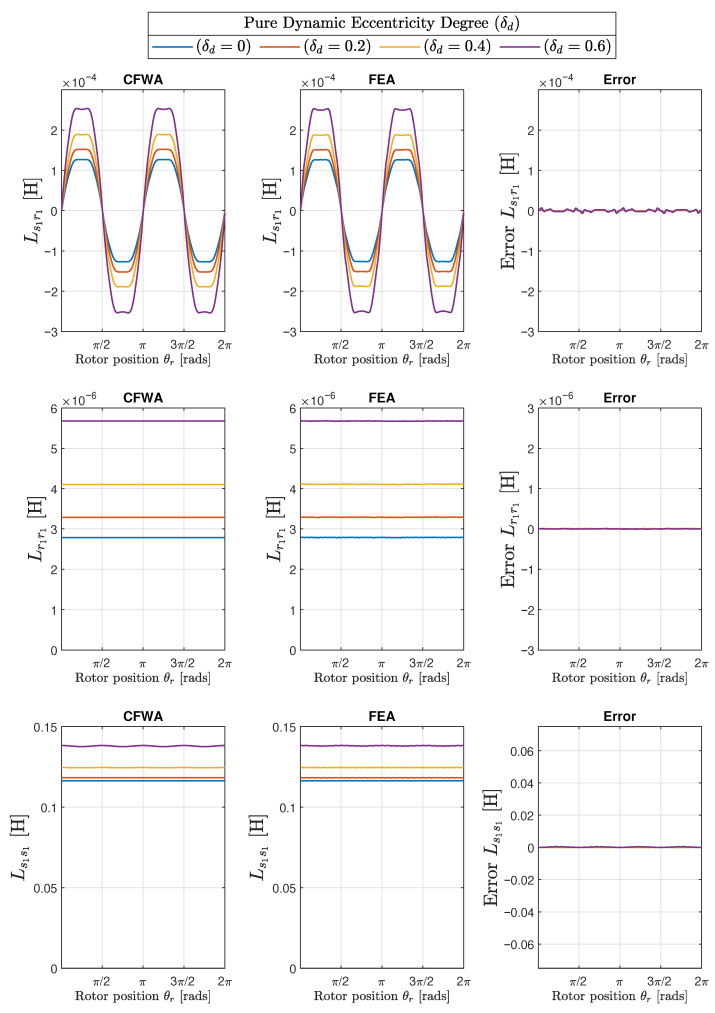
Mutual inductance between the first stator and the first rotor phase ($L_{s_{1}r_{1}}$, **top row**) and self-inductances of the first rotor phase ($L_{r_{1}r_{1}}$, **middle row**) and of the first stator phase ($L_{s_{1}s_{1}}$, **bottom row**) for the IM of Appendix A with three different degrees of dynamic ($\delta_{d}$) eccentricity: $\delta_{d}$ = 0.2, $\delta_{d}$ = 0.4, and $\delta_{d}$ = 0.6. The case of healthy machine $\delta_{d}$ = 0.0 has also been included for comparative purposes. The first column presents the results obtained with the conformal winding tensor approach, the second column presents the results obtained with FEA, and the third column presents the errors between both approaches.

**Figure 20:**
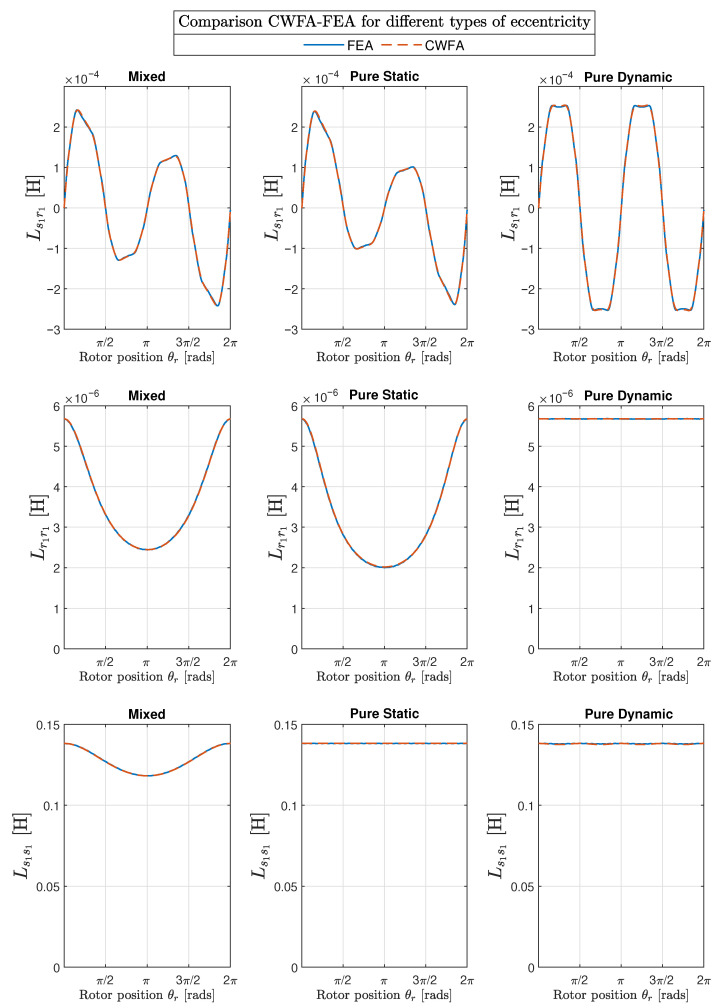
Mutual inductance between the first stator and the first rotor phase ($L_{s_{1}r_{1}}$, **top row**) and self-inductances of the first rotor phase ($L_{r_{1}r_{1}}$, **middle row**) and of the first stator phase ($L_{s_{1}s_{1}}$, **bottom row**), for the IM of Appendix A with three different types of eccentricity: mixed eccentricity ($\delta_{s}$ = 0.4, $\delta_{d}$ = 0.2) in the first column, pure static eccentricity ($\delta_{s}=0.6$) in the second column, and pure dynamic eccentricity ($\delta_{d}=0.6$) in the third column. The results obtained with the conformal winding approach and with FEA have been plotted together for comparison purposes.

The results obtained with the proposed method of the conformal winding tensor approach are compared graphically with those obtained with the FEA in the first two columns of Figure 17 (mixed eccentricity), in Figure 18 (pure static eccentricity), and Figure 19 (pure dynamic eccentricity). In addition, a third column has been added in each figure to display the differences between these two approaches. Figure 20 show superimposed results obtained with FEA and CWTA approaches, with a remarkable coincidence.

To evaluate the accuracy of the results obtained with CFWA, the root mean square error (RMSE) index has been chosen in this work, following the proposals presented in [66,67,68].

The root mean square errors corresponding to the differences between CFWA and FEA, shown in the third column of Figure 17, Figure 18 and Figure 19, have been evaluated as follows:

(33) $$\mathrm{RMSE}=\sum_{i=1}^{N}\sqrt{\frac{{\left(L_{CWFA}\left(i\right)-L_{FEA}\left(i\right)\right)}^{2}}{N}}$$

where $L_{CWFA}\left(i\right)$ is the inductance computed with the proposed method for a given rotor position *i*, $L_{FEA}\left(i\right)$ is the inductance calculated with FEA for the same rotor position *i*, and *N* is the total number of rotor positions considered in FEA simulation (N=1000). The calculated RMSE errors are displayed in Table 1 for the 10 degrees of eccentricity considered in this work (columns 1 and 2), for the mutual inductance between the first stator and rotor phases ($L_{s_{1}r_{1}}$, column 3), and for the self inductances of the first rotor phase ($L_{r_{1}r_{1}}$, column 4) and of the first stator phase ($L_{s_{1}s_{1}}$, column 5).

A direct observation of Figure 17, Figure 18, Figure 19 and Figure 20 and the results given in Table 1 show that the errors in the inductance matrix are very small and are due to the effects of change of the reluctance produced by the relative position between stator and rotor slots. This effect has not been taken into account in the conformal winding tensor approach presented in this work, although it could be included in the model using an additional conformal transformation, as in [69]. This is a point that will be addressed in future works.

## 6. Experimental Validation

**Figure 21:**
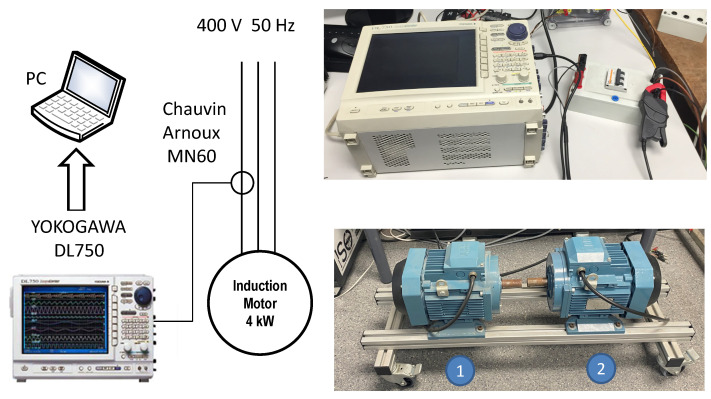
Test rig used for the experimental validation of the proposed approach. Two motors of the same type as the simulated one, labelled as (1) and (2) in the **right**, **bottom** part of the Figure, have been experimentally tested. To avoid the influence of the coupling on the eccentricity measurement, both motors have been tested uncoupled and powered directly form the mains, as shown in the schema (**left**). The current has been recorded using a Chauvin Arnoux MN60 current probe (see Appendix B) and a Yokogawa DL750 ScopeCorder (**right**, **top**), at a rate of 10 kHz during an acquisition time of 100 s, to achieve a 0.01 Hz resolution in the current spectrum. The registered data have been stored and processed with the computer platform given in Appendix C.

To validate the proposed approach, two motors of the same type than the simulated one (see Appendix A) have been experimentally tested, using the test bench displayed in Figure 21. To avoid the influence of the coupling on the eccentricity measurement, both motors have been tested uncoupled and powered directly from the mains, as shown in Figure 21, left. The current has been recorded using a Chauvin Arnoux MN60 current probe (see Appendix B) and a Yokogawa DL750 ScopeCorder (shown Figure 21, right), at a rate of 10 kHz for an acquisition time of 100 s, to achieve a 0.01 Hz resolution in the current spectrum. The registered data have been stored and processed with the computer platform given in Appendix C. The measured speed of the the motors has been 1499.5 rpm.

The diagnosis of the mixed eccentricity fault is made by analyzing the spectrum of the motor current. This type of fault generates two sideband fault harmonics around the fundamental component at frequencies given by the following:

(34) $$f_{ecc}=f_{1}\pm f_{r}$$

where $f_{1}$ is the network frequency (50 Hz), and $f_{r}$ is the mechanical rotation frequency of the rotor. For a measured speed of 1499.5 rpm and a measured frequency of the fundamental component of 50.01 Hz, (34) gives the following.

(35) $$f_{ecc}=f_{1}\pm f_{r}=50.01\pm \frac{1499.5}{60}=50.01\pm 24.99=\left[25.02\mathrm{Hz},75\mathrm{Hz}\right]$$

**Figure 22:**
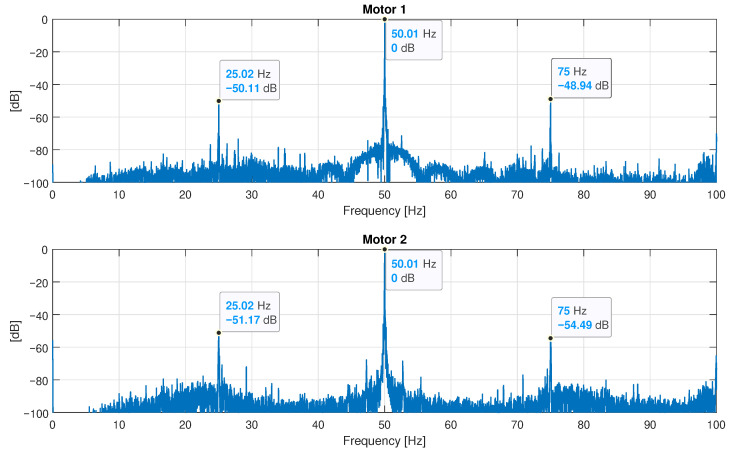
Spectra of the currents of the two tested motors, both of them of the type described in Appendix A. These spectra show the fault harmonics of an incipient-mixed eccentricity fault (marked in the figure), with a low level (around −50 dB), which may be produced by inherent and unavoidable manufacturing defects.

The spectra of the currents of both motors are shown in Figure 22. These spectra show the fault harmonics of an incipient-mixed eccentricity fault, at the exact frequencies given by (35), with a low level (around −50 dB) that can be produced by inherent and unavoidable manufacturing defects. The experimental validation is performed by simulating the motor under diverse degrees of static and dynamic eccentricity using the proposed method, obtaining the fault harmonics from the spectrum of the simulated motor current and using these results to estimate the degree of static and dynamic eccentricity of the motors.

The problem of determining the degree of mixed eccentricity using stator current analysis has been addressed in the technical literature with a time-stepping finite element method in [70,71]. In [14], an experimental index has been defined based on the geometric mean of the degree of static and dynamic eccentricity, and in [72], the amplitudes of the fault harmonics under different degrees of mixed eccentricity and load are obtained, assessing the decrease in these amplitudes with the load level. In [72], an offline method for calculating the degree of eccentricity using a standstill testing was presented. In contrast, the CWFA presented in this work allows the determination of the degree of eccentricity by direct comparison with the results obtained from a large number of IM simulations, for a wide range of both static and dynamic eccentricities.

**Figure 23:**
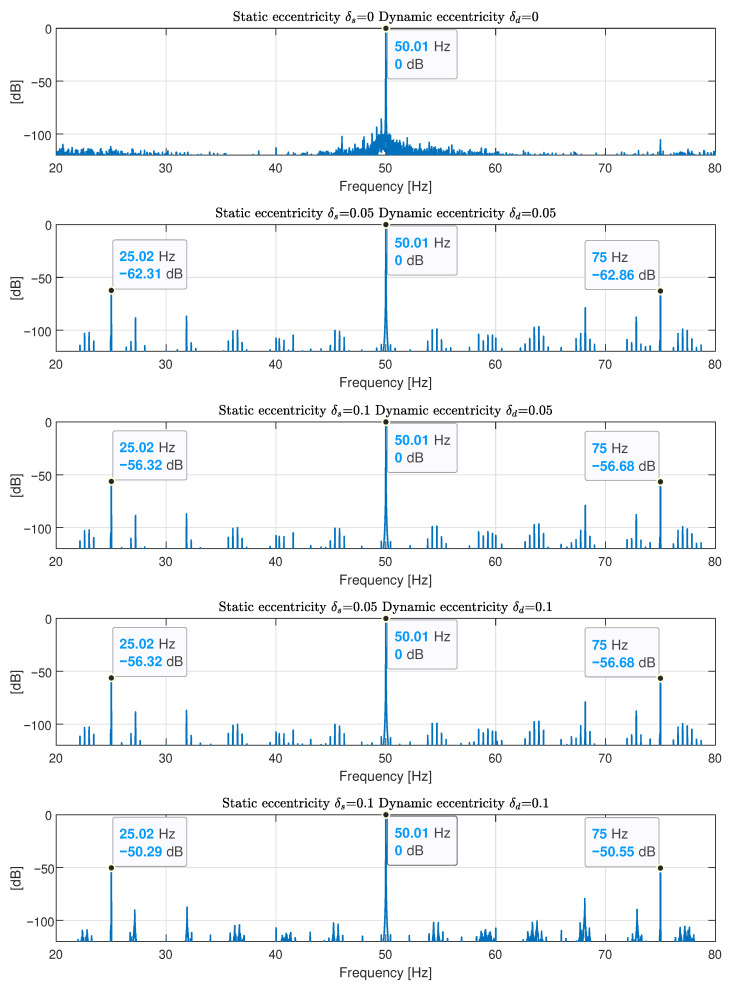
Spectra of the simulated currents of the motor described in Appendix A, for different degrees of mixed eccentricity (static eccentricity $\delta_{s}$, dynamic eccentricity $\delta_{d}$). Top: healthy motor ($\delta_{s}=0$, $\delta_{d}=0$). Below, from top to bottom, increasing mixed eccentricity faults ($\delta_{s}=0.05$, $\delta_{d}=0.05$), ($\delta_{s}=0.1$, $\delta_{d}=0.05$), ($\delta_{s}=0.05$, $\delta_{d}=0.1$), and ($\delta_{s}=0.1$, $\delta_{d}=0.1$). This last spectrum displays the fault harmonics closest to the measured ones in Figure 22, which is compatible with a degree of mixed eccentricity fault with ($\delta_{s}=0.1$, $\delta_{d}=0.1$).

The spectra of the simulated motor currents have been represented in Figure 23, for different degrees of mixed eccentricity (static eccentricity $\delta_{s}$; dynamic eccentricity $\delta_{d}$). In Figure 23, top, the spectrum of the motor in healthy conditions is displayed, without showing fault harmonics. Below, from top to bottom, the spectra of the motor current with increasing degrees of mixed eccentricity faults are displayed in Figure 23: ($\delta_{s}=0.05$, $\delta_{d}=0.05$), ($\delta_{s}=0.1$, $\delta_{d}=0.05$), ($\delta_{s}=0.05$, $\delta_{d}=0.1$), and ($\delta_{s}=0.1$, $\delta_{d}=0.1$). The amplitudes of the fault harmonics have been tabulated in Table 2, together with the fault harmonics measured in the two tested motors.

The last spectrum displayed in Figure 23, bottom, tabulated in the last row of Table 2, shows the simulated fault harmonics with an amplitude close to those measured in Figure 22, which indicates an incipient mixed eccentricity fault that is compatible with a degree of ($\delta_{s}=0.1$, $\delta_{d}=0.1$) in both motors tested.

It is worth mentioning that, as [73] states, the relative contributions of static and dynamic eccentricity to the mixed eccentricity fault cannot be separated. Therefore, the simulated machine with a mixed eccentricity of ($\delta_{s}=0.1$, $\delta_{d}=0.05$) and ($\delta_{s}=0.05$, $\delta_{d}=0.1$) generates fault harmonics with the same amplitude, as seen in their corresponding spectra (Figure 23 and their corresponding rows in Table 2).

## 7. Conclusions

The conformal transformation combined with the winding tensor approach is able to generate the inductance matrix of an induction machine with a mixed eccentricity fault, with a similar accuracy to FEA, and a much lower computation cost. Furthermore, it can be coded very simply, compared with other analytical approaches presented in the technical literature. This allows the efficient simulation of IM with a wide variety of static and dynamic eccentricity degrees, which makes it possible to develop new and advanced algorithms for fault detection, train expert systems with simulated data, or estimate the degree of the eccentricity fault on a given motor, as in this work. The conformal winding tensor approach could also be used to simulate the simultaneous presence of different types of fault (mixed eccentricity, bar breakages, inter-turn short circuits, etc.) and also to simulate the faulty machine under transient conditions. Both fields of application are a work in progress at this moment.

## Figures and Tables

**Table 1:** RMSE of the proposed conformal winding tensor compared with FEA.

Degree of Eccentricity				
Static $\delta_{s}$	Dynamic $\delta_{d}$	Error $L_{s_{1}r_{1}}$	Error $L_{r_{1}r_{1}}$	Error $L_{s_{1}s_{1}}$
0.0	0.0	1.10 × 10^−6^	2.84 × 10^−9^	3.69 × 10^−5^
0.0	0.2	1.39 × 10^−6^	3.45 × 10^−9^	3.86 × 10^−5^
0.0	0.4	1.84 × 10^−6^	5.20 × 10^−9^	6.80 × 10^−5^
0.0	0.6	2.65 × 10^−6^	2.60 × 10^−9^	2.46 × 10^−4^
0.2	0.0	1.13 × 10^−6^	4.54 × 10^−9^	3.85 × 10^−5^
0.2	0.2	1.48 × 10^−6^	5.20 × 10^−9^	4.24 × 10^−5^
0.2	0.4	2.06 × 10^−6^	6.83 × 10^−9^	1.20 × 10^−4^
0.4	0.0	1.29 × 10^−6^	7.94 × 10^−9^	4.38 × 10^−5^
0.4	0.2	1.85 × 10^−6^	9.27 × 10^−9^	8.27 × 10^−5^
0.6	0.0	1.82 × 10^−6^	1.48 × 10^−8^	5.29 × 10^−5^

**Table 2:** Amplitude of the fault harmonics corresponding to the experimental tests and the simulated motor conditions.

Motor	Eccentricity Degree		Amplitude of the Fault Harmonics	
	Static $\delta_{s}$	Dynamic $\delta_{d}$	$f_{1}-f_{r}=25.2$ Hz	$f_{1}+f_{r}=75$ Hz
Motor 1	Unknown	Unknown	−50.11 dB	−48.94 dB
Motor 2	Unknown	Unknown	−51.17 dB	−54.49 dB
Simulated	0	0	<−100 dB	<−100 dB
	0.05	0.05	−62.31 dB	−62.86 dB
	0.1	0.05	−56.32 dB	−56.68 dB
	0.05	0.1	−56.32 dB	−56.68 dB
	0.1	0.1	−50.29 dB	−50.55 dB

## Data Availability

Not applicable.

## Appendix A. Commercial IM

Three-phase IM. Rated characteristics: P=4kW, f=50Hz, U=230/400V, I=8.9/15.4A, n=1435r/min, and cosφ=0.8.

Machine dimensions: Effective length of the magnetic core = 98 mm, radius at the middle of the air gap = 57.3 mm, and air gap length = 0.4 mm.

Stator: Three-phase winding, 36 slots, 32 wires/slot, slot opening width = 3.15 mm, phase resistance 1.69 Ω, phase leakage inductance = $6.1\times 10^{-3}$ H, and Carter’s factor = 1.197.

Rotor: Squirrel-cage winding, 28 bars, slot opening width = 3 mm, skew = one slot pitch, rotor bar resistance = $9\times 10^{-5}\;\Omega$, rotor bar leakage inductance = $3.45\times 10^{-7}$ H, end ring leakage resistance = $5.53\times 10^{-6}\;\Omega$, end ring inductance = $3.68\times 10^{-8}$ H, and Carter’s factor = 1.042.

## Appendix B. Computer Features

CPU: Intel Core i7-2600K CPU @ 3.40 GHZ RAM memory: 16 GB, Matlab Version: 9.9.0.1592791 (R2020b).

## Appendix C. Current Clamp

Chauvin Arnoux MN60, Nominal measuring scope: 100 mA ..20 A; ratio input/output: 1 A/100 mV; intrinsic error: ≤2% + 50 mV; frequency use: 400 Hz to 10 kHz.
